# Bronchopulmonary dysplasia and prematurity-associated lung disease: emerging therapies to improve lifelong respiratory outcomes

**DOI:** 10.1007/s00431-026-07366-8

**Published:** 2026-09-05

**Authors:** Lorenzo Zanetto, Luca Bonadies, Sabrina Salvadori, Enrico Valerio, Elena Priante, Daniel Nardo, Victoria Niklas, Daniele De Luca, Eugenio Baraldi

**Affiliations:** 1 Department of Woman’s and Child’s Health, Study Center for Preterm Birth and Bronchopulmonary Dysplasia, University of Padova, Padova, Italy; 2 Neonatal Intensive Care Unit, Department of Woman’s and Child’s Health, Padova University Hospital, Padova, Italy; 3 Institute of Pediatric Research “Città Della Speranza”, Padova, Italy; 4 Oak Hill Bio Ltd, Altrincham, Cheshire United Kingdom; 5 Division of Paediatrics, Transportation and Neonatal Critical Care, “A.Beclere” Medical Centre, APHP-Paris Saclay University, Paris, France; 6 Pathophysiology and Therapeutic Innovation Unit, INSERM U-999, Paris Saclay University, Paris, France

**Keywords:** Bronchopulmonary dysplasia, Chronic lung disease of prematurity, Prematurity-associated lung disease, Prevention, Neonatology, Respiratory function

## Abstract

Survival of extremely preterm infants has improved owing to advances in perinatal and neonatal care. However, bronchopulmonary dysplasia (BPD) has remained unabated, and research into preventive measures for the broader spectrum of prematurity-associated lung disease (PLD) has met with limited success. We summarize emerging therapeutic strategies with the potential to favorably influence the lifelong trajectory of lung health of preterm infants, especially the most immature. This narrative review examines recent preclinical and clinical studies on therapies for BPD prevention, focusing on mechanism-based interventions, biologic strategies, and innovative translational tools. Preclinical evidence converges on actionable targets in alveolarization, inflammatory pathways, redox and mitochondrial homeostasis, vascular and matrix remodeling. Novel molecules and drug-repurposing strategies are being tested in animal models, while patient-derived platforms may enable personalized therapeutic approaches. Mechanism-based approaches are progressing through Phase 1–2 clinical trials; pending efficacy analyses, insulin-like growth factor-1 replacement may become the first pharmacological therapy specifically approved to reduce or attenuate BPD. In parallel, cell-based therapies and extracellular vesicle strategies offer a complementary paradigm, acting through pleiotropic biological reprogramming of the injured lung. Ethical-regulatory complexity and the challenges of standardizing and scaling cell-based and cell-free therapies may limit clinical translation.

*Conclusion*: A growing repertoire of therapies for BPD holds promise for modifying early respiratory illness and the long-term burden of PLD. Realizing this potential will benefit from both mechanism-informed combinatorial approaches and pleiotropic strategies, supported by patient-derived translational tools and adequately powered efficacy evaluations. Equally essential are tailored neonatal-first regulatory pathways linking the continuum of BPD to the later respiratory morbidity of PLD, a life-course condition with significant public health relevance beyond the neonatal period.

**Table:** 

**What is Known:** • *Bronchopulmonary dysplasia (BPD) is the current clinical and regulatory endpoint for neonatal pulmonary therapies*. • *BPD definitions capture the most severe manifestations of prematurity-associated lung disease (PLD), a broader continuum of respiratory morbidity reaching into adulthood; attenuating BPD may improve the whole spectrum of PLD*.	
**What is New:** • *Therapies targeting alveolar, inflammatory, oxidative, vascular, and remodeling pathways are in clinical development; pending late-stage trials, IGF-1 replacement may become the first disease-modifying therapy to reduce BPD*. • *Novel targets, experimental platforms, and repurposed drugs hold promise for precision therapies in preterm infants*.

**Supplementary information:**

The online version contains supplementary material available at https://doi.org/10.1007/s00431-026-07366-8.

## Introduction

Preterm birth remains a major global health challenge, affecting approximately 10% of live births worldwide and representing a leading cause of neonatal morbidity and mortality [1]. Advances in perinatal and neonatal care, including antenatal corticosteroids, surfactant replacement therapy, and refined respiratory support strategies, have substantially improved survival, particularly among extremely low gestational age neonates (ELGANs, born below 28 weeks of gestation). However, a survival improvement at the more severe end of the spectrum of prematurity has been accompanied by an increased burden of chronic respiratory morbidity, both in childhood and adulthood [2–5]. So far, the need for oxygen and respiratory support during the neonatal intensive care unit admission has been variably integrated to diagnose and grade bronchopulmonary dysplasia (BPD) [6, 7]. This disease entity has received the greatest epidemiological and research attention, and has long been considered the main determinant of long-term pulmonary outcome in preterm infants, thereby becoming the current clinical and regulatory endpoint for pulmonary therapies; yet it has itself gone through profound changes from its initial description to contemporary times [8, 9]. While the “classic” BPD was characterized by severe airway injury, fibrosis, and emphysematous changes, the “new” BPD reflects an arrest of lung development and is typical of extreme prematurity, where impaired alveolarization and dysmorphic pulmonary vasculature predominate over other abnormalities [10]. However, in recent years, there is growing recognition that even mild degrees of prematurity can alter lifelong respiratory health [11]. These observations underscore how respiratory disease in preterm infants is itself a multifaceted entity, shaped by the interplay of distinct injurious exposures. This argues for moving beyond a purely neonatal definition of BPD toward endotypes and clinical phenotypes linked, across the lifespan, to healthcare needs, prolonged oxygen therapy, recurrent hospitalization, wheezing disorders, pulmonary hypertension, and the adult development of early-onset chronic obstructive pulmonary disease (COPD)-like phenotypes [12]. For these reasons, the framework of prematurity-associated lung disease (PLD) has been conceptualized to collectively describe the heterogeneous respiratory consequences of prematurity from birth to adulthood and to unify efforts towards healthier respiratory outcomes for all preterm infants [13].

Importantly, PLD can present with a range of multisystem comorbidities; these conditions reflect overlapping perinatal insults and shared pathogenic pathways, with adverse consequences on overall health that may translate into a substantial individual and societal burden of morbidity as these patients reach adulthood and older age [14, 15]. Interventions targeting lung protection, inflammation, oxidative stress, vascular development, and tissue regeneration may have lasting effects on lung structure and function, and potentially extend their benefit to other organ systems involved through shared mechanisms. However, while many therapies show promise in reducing early respiratory morbidity, robust evidence demonstrating sustained improvements in long-term outcomes remains limited. Notably, targeted single-axis interventions have not translated into clear benefit [16]. Taken together, these observations call for strategies addressing the full mechanistic complexity of PLD, with the potential to reshape the respiratory developmental course of preterm-born individuals.

This review aims to summarize emerging treatments in preterm infants that may influence long-term pulmonary outcomes. By integrating pathophysiological insights with clinical evidence, we seek to identify which interventions have the potential not only to improve survival and short-term respiratory stability but also to favorably modify the lifelong trajectory of lung health in this vulnerable population.

## Pathophysiological background

Lung development proceeds through highly regulated stages-embryonic, pseudoglandular, canalicular, saccular, and alveolar-culminating in the coordinated development of alveolar structures and the pulmonary capillary network required for efficient gas exchange. Extremely preterm birth typically occurs during the late canalicular or early saccular stage of lung development, when alveolarization and microvascular maturation remain incomplete [10, 17]. Prenatal factors, such as intrauterine growth restriction, may already disrupt critical developmental processes during fetal life and are frequently associated with preterm birth itself. Acting on this immature lung, many possible extrauterine noxae can trigger additional pathogenetic pathways [13].

While respiratory support is often lifesaving in preterm infants, ventilation and supplemental oxygen can amplify lung injury. Ventilator-induced lung injury (volu-baro-atelecto- and bio-trauma) can activate inflammatory cascades that interfere with alveolar and vascular maturation [18]. Similarly, hyperoxia promotes oxidative stress due to immature antioxidant defenses, leading to epithelial and endothelial damage and disruption of growth factor signaling [19]. Inflammation represents a unifying mechanism linking mechanical and oxidative injury. Proinflammatory cytokines that impair septation and angiogenesis, lack of surfactant, inefficient antioxidant mechanisms, lower compliance, and inadequate fluid clearance may perpetuate a vicious cycle of persistent early inflammatory activation that contributes to airway remodeling and long-term airflow limitation [20]. Beyond these pathways, three interconnected axes of extrapulmonary influences target the developing lung. The first is the gut-lung axis: early antibiotic exposure and gut dysbiosis appear to shape inflammation and immune training, with consequences for BPD risk in observational data [21–24]. The second is neuro-pulmonary signaling. Mechanical ventilation, repeated airway management, and hyperoxia appear to sensitize vagal afferents, and the resulting cholinergic drive modulates pulmonary inflammation and airway innervation. This frontier remains hypothesis-generating and awaits preclinical confirmation [25]. The third is metabolic reprogramming: perinatal insults skew nutrient sensing through mTOR and AMPK and shift substrate utilization; metabolic signatures include mitochondrial dysfunction, oxidative stress, and altered purine and amino acid metabolism, and remain detectable into adulthood [14, 26, 27]. Nutrient availability intersects this last axis directly. Nitric oxide promotes vasodilation, angiogenesis, and alveolarization; with preterm infants frequently suffering arginine deficiency, its endogenous synthesis is limited in this population [28]. Together, these factors highlight how early exposures can permanently alter lung development and influence lifelong respiratory trajectories.

## Emerging treatments

### Insulin-like growth factor-1 (IGF-1)

Insulin-like growth factor-1 (IGF-1) is the principal fetal growth factor supporting coordinated development of lung, brain, and eye during the last trimester of pregnancy. It is supplied predominantly through the placenta until approximately 30 weeks of postmenstrual age (PMA), when the fetal liver progressively acquires the capacity to produce it [29, 30]. In the developing lung, IGF-1 drives alveolar epithelial proliferation and differentiation and induces vascular endothelial growth factor (VEGF) transcription, thereby coupling alveologenesis to microvascular growth, while attenuating transforming growth factor-β (TGF-β) signaling and the fibroproliferative response to neonatal injury [29, 31]. Preclinical evidence confirms the essential nature of the IGF-1 axis as a driver of lung development: IGF-1R-null mice suffer pulmonary hypoplasia and die of respiratory failure [32]. Despite strong evidence for inflammatory involvement in PLD, the fundamental deficit in preterm lung disease may lie in interrupted development: alveolarization, microvascular growth, and maturational trajectories are truncated at birth, often further disrupted by antenatal insults [33]. Accordingly, anti-inflammatory strategies per se—including budesonide/surfactant and azithromycin trials—have yielded disappointing results, underscoring that effective therapies may need to actively stimulate lung growth rather than simply modulate its inflammatory sequelae [16, 34].

Extreme prematurity disrupts placental endocrine function at a particularly vulnerable window. Circulating within days, IGF-1 falls to approximately one-fifth of fetal levels and remains below the physiological intrauterine range for weeks [35]. The deficit is amplified in ELGANs by the immature liver’s inability to assemble the ternary IGF-1/IGF binding protein-3/acid-labile-subunit complex, which normally prolongs IGF-1 bioavailability; the half-life of free IGF-1 drops to less than 1 h, so that physiological replacement requires continuous intravenous infusion [29]. Low postnatal IGF-1 has been consistently associated with subsequent BPD, retinopathy of prematurity, intraventricular hemorrhage and impaired somatic and brain growth [30].

In preclinical models of pre-eclampsia, chorioamnionitis and hyperoxia, recombinant human IGF-1 combined with its binding protein (IGFBP3) produced dose-dependent improvements in alveolar count, microvascular density and lung compliance, and prevented right ventricular hypertrophy [29, 32]. In mechanically ventilated preterm lambs delivered at approximately 128 days' gestation (equivalent to ≈28 weeks in humans), a 7-day continuous infusion improved gas exchange, enhanced alveolar and capillary development, upregulated VEGF-receptor, and reduced terminal bronchiolar smooth-muscle accumulation, without hepatic, renal, or hemodynamic toxicity [35].

Clinically, the Phase 2a trial of OHB-607 (IGF-1/IGFBP3 complex) missed its primary ophthalmological endpoint but a secondary analysis reported a 53% relative reduction in severe BPD (89% in the evaluable set that reached the target physiological exposure of 28–109 µg/L, a subgroup defined by achieved exposure rather than by randomization) [30, 36]. These findings shifted the clinical development of OHB-607 toward BPD prevention and provided the rationale for the multicenter Phase 2b trial (NCT03253263), which completed randomization of 338 infants born at 23⁺⁰–27⁺⁶ weeks to continuous infusion of OHB-607 until 29⁺⁶ weeks PMA versus standard care [30]. By acting during the canalicular-to-saccular window when distal airspaces and the alveolar capillary bed are simultaneously formed, IGF-1 replacement may preserve the structural ceiling against which lifelong lung function develops. Recruitment is complete. Should the efficacy analyses prove favorable, IGF-1 replacement could become the first pharmacological therapy specifically approved to reduce the incidence or severity of BPD. Whether this translates into measurable improvements in spirometric outcomes and PLD-related morbidities during adolescence and adulthood will require the long-term follow-up of this trial and future longitudinal cohorts.

### Mesenchymal stromal cells (MSCs)

Mesenchymal stromal cells (MSCs) have emerged as a promising therapeutic strategy for BPD because of their pleiotropic effects. Rather than engrafting and differentiating into lung tissue, MSCs appear to exert most of their therapeutic impact through paracrine mechanisms, releasing a wide range of bioactive molecules (cytokines, growth factors, and extracellular vesicles (EVs)) that modulate macrophage-mediated inflammation, promote tissue repair, reduce fibrotic reactions and oxidative stress, protect surfactant, while supporting lung alveolar and vascular development [37–39]. Beyond their respiratory effects, MSCs may also exert broader systemic benefits in preterm infants, with emerging preclinical and early clinical evidence suggesting potential effects on neurodevelopmental injury, intestinal inflammation, and sepsis-related complications [40–43]. Among the possible sources, a substantial body of preclinical evidence supports the human umbilical cord (UC) or UC-blood derivation for BPD. MSC populations are functionally heterogeneous, with implications for their therapeutic application in BPD. Single-cell RNA sequencing revealed distinct transcriptional clusters with divergent biological properties, including progenitor-like and fibroblast-like subpopulations, and the transcriptomic profile of MSCs has been shown to correlate with their regenerative efficacy [44, 45].

Phase I trials have demonstrated that administering allogeneic UC-derived MSCs in ELGANs at high risk of BPD is feasible and appears safe, with no major treatment-related adverse events reported [39, 46–48]. To date, only one phase II study has further explored the clinical application of MSC therapy (NCT01828957), though not powered for efficacy [41]. At 5-year follow-up (NCT01897987), mortality, growth, and neurodevelopmental outcomes did not differ between groups, and a non-significant trend towards lower respiratory morbidity was observed in the treated group [49]. Notably, this remains the only clinical program in this field to have reported respiratory outcomes well beyond infancy.

Several challenges remain before MSC-based therapies can be widely implemented. Open questions include the standardization of cell manufacturing, pre-injection conditioning, functional and quality-control testing, donor and tissue selection, dosing strategies, timing and route of administration, patient selection, and outcome measures. The functional heterogeneity noted above probably underlies much of the variability across preclinical studies, making the selection or enrichment of subpopulations with superior reparative capacity a central translational goal [39, 50].

### Extracellular vesicles

EVs are nanosized lipid-bilayer particles of 30–5000 nm, carrying a heterogeneous cargo of proteins, lipids, mRNAs, regulatory RNAs, and even mitochondria, and mediating intercellular communication both locally and at a distance [51]. EVs released by MSCs (MSC-EVs) are now considered the effectors of their paracrine therapeutic activity and recapitulate most of their regenerative properties (combining anti-inflammatory, pro-angiogenic and anti-fibrotic effects), while overcoming practical barriers to implementation, like storage and large-scale manufacturing, as well as theoretical safety limitations of live-cell therapy such as engraftment, immunogenicity, and phenotypic instability under inflammatory stress [52, 53].

In the preterm lung, EVs are detected from 22 weeks of gestation in tracheal aspirates and amniotic fluid, and their size and surface markers change across the canalicular and saccular stages, suggesting a possible involvement in developmental processes [54]. EV profiles diverge in those who go on to develop BPD. EVs recovered from their tracheal aspirates are smaller and display an altered immune/epithelial surface-marker signature, with depletion of protective microRNAs. By contrast, circulating EVs from hyperoxia-exposed rat pups carry alveolar epithelial proteins and pyroptosis-related mediators, contributing to concomitant lung and brain damage [54]. Intravenous, intraperitoneal or intratracheal MSC-EVs in neonatal rodents exposed to hyperoxia consistently improve alveolarization, reduce septal fibrosis, restore vascular density, attenuate pulmonary hypertension and shift pulmonary macrophages towards an M2 anti-inflammatory phenotype, with the cardiopulmonary benefit of a single neonatal dose persisting into young adulthood [55–59]. Antenatal MSC-EVs preserve lung development in chorioamnionitis models [60]; multifactorial injury combining endotoxin, mechanical ventilation, and hyperoxia is mitigated by intratracheal EVs with concurrent neuroprotection [61]; and—closer to the clinical context—intravenous bone-marrow MSC-EVs improve gas exchange, alveolar–capillary growth, and feeding tolerance in mechanically ventilated preterm lambs [62].

The EVENEW Phase I/II trial (NCT06279741) of intratracheal allogeneic UC MSC-derived EVs (EXOB-001) in ELGANs is currently recruiting in Europe and represents the first regulatory milestone for a cell-free therapy in BPD. Standardization of isolation, characterization, dosing and pharmacokinetics, together with rigorous adherence to Minimal Information for Studies of Extracellular Vesicles (MISEV) reporting guidelines, remains the principal hurdle before EVs can meaningfully reshape the life-course trajectory of lung function in extreme prematurity.

### Surfactant protein-D (SP-D)

Surfactant Protein-D (SP-D), together with SP-A, belongs to the collectin family of innate immunity proteins and is synthesized by alveolar type II cells and club cells. Unlike the hydrophobic surfactant proteins, SP-D has minimal biophysical activity but is a central modulator of pulmonary inflammation and innate host defense [63]. SP-D opsonizes bacteria, viruses, and fungi, attenuates toll-like receptor 4 (TLR4)/nuclear factor kappa B (NF-κB) and NOD-like receptor family pyrin domain-containing 3 (NLRP3)-inflammasome signaling, downregulates pro-inflammatory cytokine release, limits matrix metalloproteinase-2/9 (MMP-2/9) production by alveolar macrophages, and inhibits neutrophil extracellular trap formation [63]. SP-D also contributes to surfactant lipid homeostasis and to the regulation of postnatal surfactant pool sizes.

SP-D’s immunomodulatory activity depends on its assembly into higher-order oligomeric structures, with smaller fragments retaining only partial function [63]. In preterm infants who develop BPD, both the amount and the assembly of bronchoalveolar SP-D appear impaired: levels measured in the first hours of life rise with gestational age, are significantly lower in those who later develop the disease [64], and are skewed towards poorly active low-oligomeric forms [65]. SP-D is induced by chorioamnionitis [64], a condition that itself raises BPD risk and is associated with high secretory phospholipase A2 (sPLA2) activity [66, 67]. Consistent with the role described above, SP-D acts as a counter-regulatory response that protects only when adequate: higher early levels accordingly correlate with shorter invasive ventilation and hospital stay [68], whereas sPLA2 activity rises with longer ventilation [64, 69]. Certain SP-D polymorphisms further link the protein to respiratory distress at birth, oxygen supplementation and respiratory support, indicating a constitutive contribution [70]. Serum SP-D in the first week of life has not consistently mirrored these bronchoalveolar findings [71], confirming that the alveolar compartment is the relevant biological space.

Preclinical evidence supports a therapeutic role for SP-D replacement. In mechanically ventilated premature lambs, intratracheal recombinant human SP-D added to commercial surfactant reduced total inflammatory cells, neutrophil elastase activity, and interleukin-8 in the lung compared with surfactant alone; in neonatal rodent models of hyperoxia and endotoxemia, exogenous SP-D attenuated pro-inflammatory cytokine release, oxidative injury, and alveolar damage [63].

These observations have now reached early clinical testing. The first-in-neonates Phase 1b randomized trial of zelpultide alfa (recombinant human SP-D) enrolled 37 extremely preterm infants and showed that daily intratracheal administration for up to 7 days was safe and well-tolerated, with no dose-limiting toxicities; preliminary efficacy indicators included a lower incidence of BPD and a shorter duration of mechanical ventilation compared with air-sham, alongside a higher mortality in the treated arm; the study was not powered for efficacy [64]. These findings have prompted the ZELA trial (NCT06897839), a randomized, double-blind, placebo-controlled Phase 2b/3 study currently recruiting neonates born at 22⁺⁰–27⁺⁶. It is too early to assess whether early SP-D replacement will durably modify the diseased trajectory underlying PLD, but the convergence of mechanistic biology, biomarker data, preclinical signals, and first-in-human evidence makes it a credible candidate.

### Anakinra

Interleukin-1 (IL-1α and IL-1β) sits upstream of the inflammatory cascade sustaining BPD. IL-1β activates NF-κB and the NLRP3-inflammasome, amplifies tumor necrosis factor (TNF)-α, IL-6, and IL-8, recruits and primes monocytes, and links lung injury to the parallel inflammatory damage of brain and gut that defines extreme prematurity [72]. In preterm infants, tracheal-aspirate IL-1β is elevated days before the clinical onset of BPD, and correlates with the duration of mechanical ventilation and supplemental oxygen, particularly when Ureaplasma colonization is present [73].

The pharmacological case for anakinra is unusual in the BPD landscape because the drug already exists, making it a candidate for repurposing rather than de novo development. Recombinant human IL-1 receptor antagonist (IL-1Ra, anakinra) has been used in pediatric and adult inflammatory diseases for more than two decades—including in neonates with CINCA/NOMID syndrome—with a well-characterized safety, pharmacokinetic, and efficacy profile [72]. In murine models combining perinatal LPS and postnatal hyperoxia, daily IL-1Ra prevents the alveolar simplification, septal disorganization and gas-exchange impairment of experimental BPD [74], attenuates airway remodeling [75], and preserves pulmonary vascular density while limiting the rise in pulmonary vascular resistance characteristic of BPD-associated pulmonary hypertension [76].

Clinical translation is led by the investigator-initiated Anakinra Pilot trial (NCT05280340), an open-label Phase I/IIa dose-escalation study in 24 infants born at 24⁺⁰–27⁺⁶ weeks, administering intravenous anakinra over the first 21 days of life [72]. The trial has completed enrolment and is currently in the follow-up phase, with results awaited; no prospective clinical efficacy data are therefore yet available in this population. Meanwhile, De Rose et al. reported promising respiratory outcomes in a retrospective neonatal cohort, a hypothesis generating observation. However, only 4 patients were ELGANs with BPD (the population most at risk and targeted by the ongoing trial), underscoring the continued need for trial data in this specific population [77].

### From the bench: emerging preclinical therapies

**Fig. 1:**
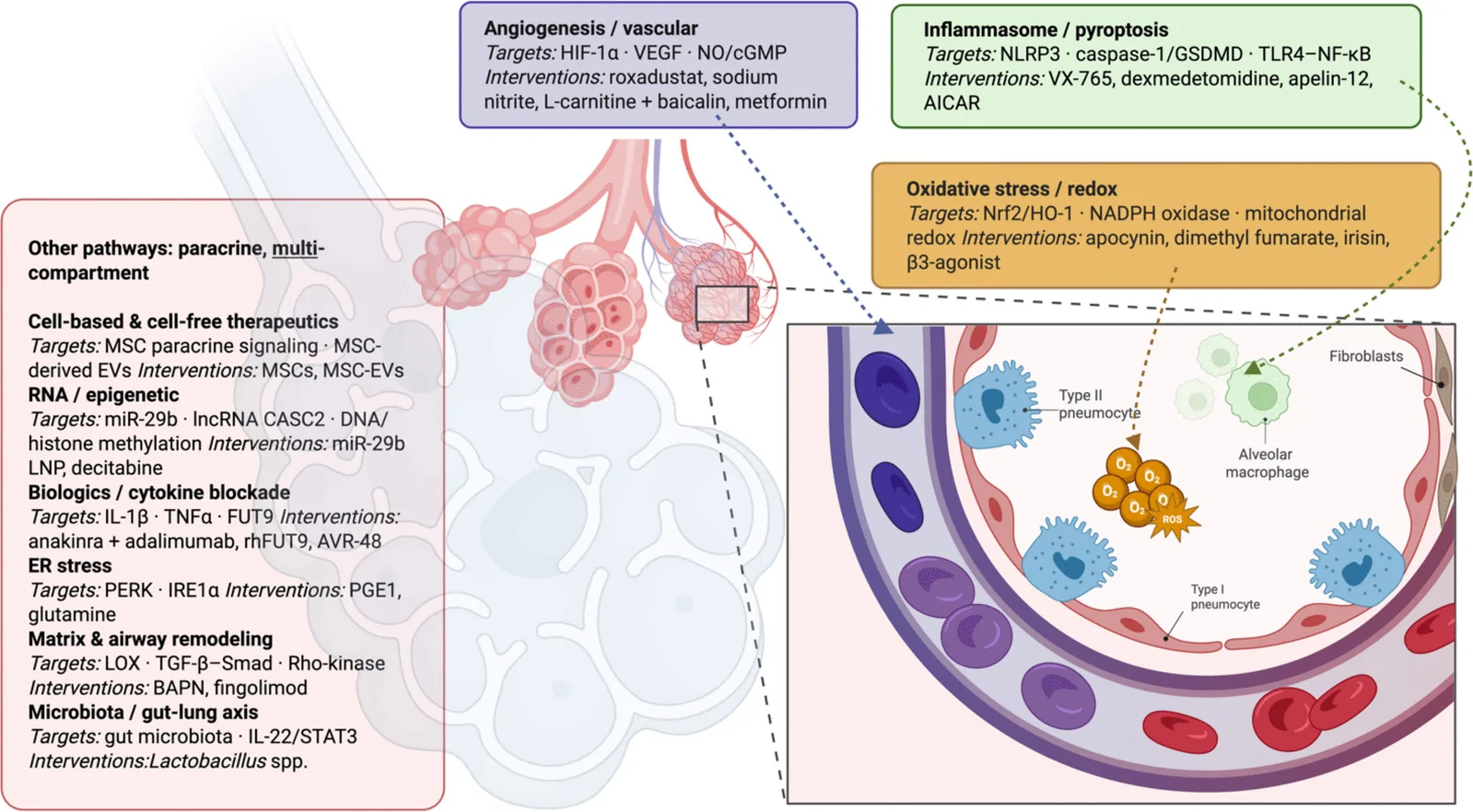
Preclinical targets of translational research in animal models of bronchopulmonary dysplasia from the last 5 years. Created in BioRender. Zanetto, L. (2026) https://BioRender.com/7lqdafp

Preclinical studies from the last 5 years in experimental models of BPD/PLD are uncovering a range of mechanistically distinct but potentially complementary therapeutic targets (Table 1, Fig. 1). Beyond descriptive pathology, these approaches focus on actionable biological processes. These could be broadly grouped into inflammasome-driven and NF-κB-mediated inflammation, oxidative and mitochondrial stress, and the vascular/angiogenic axis. Additional themes include endoplasmic reticulum stress, matrix and airway remodeling, gut–lung microbiota modulation, and epigenetic/RNA-based strategies, illustrating an increasingly multi-compartmental view of BPD pathogenesis (Fig. 1, Table 1).

**Table 1:** Preclinical studies from the last 5 years of novel interventions in in vivo translational models of bronchopulmonary dysplasia/prematurity-associated lung disease, organized by key biological mechanisms. Studies are grouped by predominant mechanistic targets

Author, year	Model	Drug/intervention	Studied signaling pathway(s)	Key outcomes
**Section 1: inflammasome, pyroptosis, and NF-κB signaling**				
León Silva et al., 2025 [82]	Neonatal rat, hyperoxia	VX-765 (caspase-1 inhibitor)	Caspase-1/IL-1β/IL-18/GSDMD; pyroptosis	Improved alveolar structure and pulmonary vascular density; ↓ vascular remodeling, right ventricular hypertrophy, aortic stiffness and cardiac fibrosis
Feng et al., 2026 [83]	Neonatal mouse hyperoxia + AECII hyperoxia	Dexmedetomidine	PINK1/Parkin-mediated mitophagy → NLRP3 inflammasome/pyroptosis	↓ lung injury, NLRP3 activation, cytokines and pyroptosis; preserved mitochondrial integrity
Zhang et al., 2026 [84]	Neonatal mouse, hyperoxia	Betaine	FOXO1–NLRP3 axis; macrophage pyroptosis	↓ macrophage pyroptosis and inflammation; ↑ alveolar development
Yang et al., 2025(a) [85]	Neonatal rat + AECII	Nesfatin-1	HMGB1/TLR4/NF-κB/NLRP3	↓ inflammation, fibrosis, apoptosis, neutrophil influx in BALF; improved lung injury
Zhang et al., 2024(a) [86]	Rat (prenatal LPS) + placenta	Inhaled hydrogen (H₂)	TLR4–NF-κB–IL-6/NLRP3; placenta–lung axis	↓ placental inflammation (IL-6, IL-1β, IL-18); improved neonatal lung development, ↑ survival
Hao et al., 2025 [87]	Neonatal rat, prolonged hyperoxia	High-dose (50 mg/kg/die dose) Nintedanib	NF-κB; IL-1β/CXCL-1/MCP-1; apoptosis	↓ histological injury (↑ RAC/↓ MLI), ↓ apoptosis, ↓ inflammation; ↓ NF-κB. No unplanned mortality reported (see Ding et al., 2025)
**Section 2: oxidative stress and redox signaling**				
Aslan et al., 2023 [88]	Neonatal rat, hyperoxia	Prophilactic molsidomine	NO donor; oxidative stress; TNF-α/IL-1β; apoptosis	↓ histological injury, macrophage infiltration, oxidative/inflammatory stress, ↓ apoptosis
Deng et al., 2022 [89]	Neonatal rat + AECII hyperoxia	CGRP (calcitonin gene-related peptide)	Notch1/Hes1/HERP; oxidative stress (MDA/SOD)	↑ AECII viability; ↓ AECII➝ AECI transdifferentiation➝ preserved AECII progenitor pool; improved epithelial regeneration
Guzmán-Navarro et al., 2021 [90]	Rat, hyperoxia–hypoxia	Prenatal indole-3-carbinol	AhR➝ antioxidant genes (Cyp1a1, Nqo1, Gsta1); ↑ NF-κB (immunomodulatory)	↑ RAC (partial); ↓ fibrosis (early timepoint); ↓ lung injury/inflammatory infiltrates
Montgomery et al., 2026 [91]	Neonatal C57BL/6N mouse, hyperoxia	Intranasal FAD (flavin adenine dinucleotide)	FAD-glutathione reductase-GSH/GSSG redox axis	Improved redox potential (GSH/GSSG ratio); ↑ RAC ↓ MLI and ↓BALF neutrophils, ↑ BALF macrophages
Pini et al., 2026 [92]	Neonatal rat, strong hyperoxia	β3-adrenoceptor agonist at three doses (BRL37344)	β3-adrenoceptor signaling; TGF-β; VEGF/VEGFR2; fibroblast/Circulating Endothelial Progenitor Cells	↑ survival, alveolarization, lung volume; ↓ TGF-β ↓ fibrosis and oxidative stress; ↑ vascular density, VEGF and Circulating Endothelial Progenitor Cells. Protective at 3 mg/kg; 6 mg/kg ↑ mortality under hyperoxia
Reçica et al., 2024 [93]	Rat pups, > 95% O₂	Prophilactic resveratrol	NO; SOD/GPx; TNF-α/IL-1β	↓ airway hyperreactivity; restored relaxation; ↑ antioxidant defenses; ↓ inflammation; improved lung histology
Zhao et al., 2023 [94]	Neonatal mouse (males only), hyperoxia	Irisin	Nrf2/HO-1 pathway; oxidative stress	↓ MDA ↑GSH; ↑ angiogenesis and alveolarization (↑ RAC ↓ MLI)
Chu et al., 2023 [95]	Premature rat, hyperoxia	Erythromycin	GSH; TNF-α/IL-1β	↑ GSH; ↓ TNF-α/IL-1β; attenuated histologic lung injury (qualitative)
Ozdemir et al., 2022 [96]	Neonatal rat, hyperoxia	Apocynin	SOD/GSH/GPx/oxidative stress; TNF-α/IL-1β	↓ histopathological injury, oxidative stress and ↓TNF-α and IL-1β, ↓ alveolar macrophage scores; ↓ TUNEL + apoptotic cells
Yang et al., 2023 [97]	Neonatal rat + AECII, hyperoxia	Nesfatin-1	SIRT1/PGC-1α pathway; oxidative stress	↑ cell viability; ↓ apoptosis (↓ Bax, ↑ Bcl-2); ↓ ROS, ↓ pulmonary MDA, ↑ pulmonary SOD; improved alveolarization in vivo: (↑ RAC, ↓ MLI)
**Section 3: angiogenesis and vascular development**				
Chang et al., 2022 [98]	Fetal pulmonary endothelial cells, hyperoxia + neonatal mouse, hyperoxia + neonatal mouse Cpt1a-KO, hyperoxia	L-carnitine + baicalin	CPT1a/fatty acid oxidation; endothelial angiogenesis/vascular development	↓ endothelial apoptosis (↓ caspase-3); ↑ endothelial migration/tube formation; ↓ alveolar and vascular simplification (↑ RAC, ↑ vWF)
Ding et al., 2025 [99]	Neonatal rat, hyperoxia	Dose-ranging study with intraperitoneal nintedanib	Src Tyrosine kinase; IL1, TNF-α, Caspase-3	Narrow therapeutic window (low dose; lethal at a 50 mg/kg/die dose); Src phosphorylation unchanged (Src-independent mechanism by nintedanib), Restored RAC/MLI; ↑ pulmonary vascular density; ↓ right ventricular hypertrophy; ↓ vessel wall thickness; ↓ IL-1α, ↓ TNF-α, ↓ Caspase-3
Huang et al., 2021 [100]	Neonatal mouse, hyperoxia	Roxadustat	HIF-1α stabilization; VEGF; eNOS; mTOR/HIF-1α	↑ pulmonary angiogenesis (↑ VEGF, eNOS, vWF, vessel density); ↓ MLI; ↑ survival/body weight; ↑RAC
Xiang et al., 2022 [101]	Neonatal mouse, hyperoxia + RAW264.7 macrophages, hyperoxia	Metformin, purmorphamine (Shh agonist)	Shh/Gli1; M1 → M2 macrophage polarization; VEGF, TNF-α, CD31	↑ M2 macrophages; ↓ TNF-α/iNOS; ↑ angiogenesis (CD31/VEGF); improved vascular development. No effect on secondary septa. Purmorphamine reversed metformin effects on M2 polarization and VEGF
Daniel et al., 2025 [102]	Neonatal rat, hyperoxia, normoxia & intermittent hypoxia (H-IH) + acute hypoxic challenge (12 wk)	Sodium nitrite vs. iNO 10 ppm	NO/sGC/cGMP signaling; S-nitrosylation by proteomics, xantine oxidase activity; irreversibile nitration	NaNO₂: prevented all H-IH-induced abnormalities (alveolar surface area, alveolar number, MLI, septal thickness, RVH, medial wall area); restored NO and cGMP; prevented nitration; benefits persisted into adulthood (12 wk) with improved SpO₂ in acute hypoxic challenge. iNO 10 ppm: prevented PH but had minimal effects on alveolar morphology and did not prevent nitration. iNO in normoxic controls caused alveolar hypoplasia, decreased NOx and cGMP, and increased nitration (evidence of iNO toxicity at a clinical dose on the developing lung)
**Section 4: epigenetics, miRNA, and lncRNA**				
Sugar et al., 2021 [103]	Double-hit murine model (prenatal intraamniotic LPS + neonatal hyperoxia)	miR-29b delivered via lipid nanoparticles	Epigenetics: histone methylation (e.g. H3K4me3, H3K27me3, H4K20me3, PRMT1/PRMT5)	↓ septal thickness; ↓ αSMA/MMP-9; ↑ PDGFα/PDGFRα; partial/full restoration of methylation patterns; partial restoration of PRMT1; no significant improvement in terminal airspaces or alveolar area
Ji et al., 2021 [104]	Neonatal mouse, short-exposure hyperoxia + BEAS-2B cells, hyperoxia	Adenoviral vector overexpressing lncRNA CASC2; rescue with miR-194-5p agomiR or sh-CAV1	CASC2–miR-194-5p–CAV1 axis; TGF-β1, CD31	↓ TGF-β (cytoplasmic CASC2 competitively binds miR-194-5p, derepressing CAV1, leading to TGF-β1 pathway inactivation; causality confirmed by full reversal of protection with miR-194-5p agomiR or sh-CAV1) ↓ apoptosis (TUNEL); ↑ angiogenesis (CD31), ↑ proliferation (ki67); attenuated lung injury
Heyob et al., 2023 [105]	Double-hit murine model (prenatal intraamniotic LPS + neonatal hyperoxia)	DNMT inhibitors: decitabine, RG108	DNA methylation; DNMT; TGF-β1/p-SMAD2/3 signaling; surfactant protein C	Decitabine: modest improvement in alveolarization; ↓ p-SMAD2/3; ↑ surfactant protein C. RG108: no evident benefit
**Section 5: cell-based therapies and biologic agents**				
Song et al., 2026 [106]	Neonatal rat, prolonged hyperoxia	Rat bone marrow-derived MSCs overexpressing PRDX6, generated by lentiviral PRDX6-GFP transduction	PRDX6–MANF secretion (paracrine/autocrine); VEGF/CD31/vWF	↑ BMSC lung recruitment; ↑ MANF in lung/BALF/serum ↓ apoptosis (caspase-3); ↓ TNF-α/IL-6/IL-1β; ↑ angiogenesis (VEGF/CD31); ↓ vascular remodeling
Chaubey et al., 2021 [107]	Neonatal mouse, hyperoxia; human RDS/BPD lung tissues for SSEA-1 expression comparison	Recombinant human FUT9	SSEA-1/FUT9; lung stem/progenitor cells	Hyperoxia: ↓ SSEA-1, ↓ BALF total cells/neutrophils FUT9 injection: ↓ BALF protein leak, ↓ TUNEL cell death, ↑ Ki67 + proliferation; no significant reduction in lung parenchymal IL-6/IL-1β and no reversal of PH/RVH
Toth et al., 2022 [108]	Rhesus macaque, chorioamnionitis (intra-amniotic LPS)	Combined anti-IL-1β (anakinra) + anti-TNFα blockade (adalimumab) administered before LPS	IL-1/TNF–NF-κB; alveolar signaling (VEGF, PDGF, WNT); CCL/CXCL inflammatory chemokine signaling	Normalized lung injury score/septal numbers, preserved AT1–alveolar capillary interactions, restored epithelial/endothelial cellular quorum, blunted inflammatory activation
**Section 6: mixed/multi-pathway interventions**				
Xu et al., 2026 [109]	Neonatal mouse, hyperoxia from postnatal day 8	Apelin-12	Nrf2/↑HO-1; NF-κB; NLRP1 inflammasome	↓ IL-1β, IL-18; ↓ oxidative stress; improved lung function and alveolar architecture. Paradoxical suppression of NLRP1 by hyperoxia; cytokine release persisted despite NLRP1 suppression, implicating non-canonical inflammasome-independent pathways
Graumuller et al., 2026 [110]	Neonatal mouse, hyperoxia	Dimethyl fumarate	Nrf-2, paradoxical ↓ HO-1, ↓ TGF-β–Smad2 and NF-κB	Improved lung histology, ↑ cell proliferation, ↑ angiogenesis (vessel count)
Tayman et al., 2021 [111]	Neonatal rat, intra-amniotic LPS + postnatal hyperoxia	Apocynin	NADPH oxidase; Nrf2, ROS and antioxidant axis; caspase1/3, NLRP3 inflammasome, inflammatory cytokines	↑ survival; improved alveolarization (↑ RAC, ↓ MLI) and ↑ surfactant protein B/C + pneumocytes; ↓ histopathological injury score and fibrosis; coordinated ↓ of oxidative stress and ↑ of antioxidant capacity; ↑ Nrf2; ↓ NLRP3, caspase-1, caspase-3 and pro-inflammatory cytokines; ↓ neutrophil infiltration (MPO)
Das et al., 2021 [112]	Neonatal mouse, BPD + experimental PH	AVR-48 (chitin derivative)	TLR4; M2 macrophage polarization; NF-κB/TNF-α/IL-1β/TGFβ inflammatory signaling; SP-C, VEGF, Ang2, eNOS, VEGF-D	↓ NF-κB/TNF-α/IL-1β/TGFβ inflammatory signaling ↓ BALF total cells/protein; reduced apoptosis (↓ TUNEL + ↓ caspase-3); ↑ IL-10; normalized neutrophil/macrophage balance; ↑ SP-C + ATII cells’ proliferation (↑ Ki67 +, PCNA); ↑ RAC; ↑ VEGF, VEGF-D, Ang2, eNOS ↓ septal thickness,; ↑ vWF, ↓ Ang2, ↓ Fulton’s Index/RV hypertrophy
Soni et al., 2023 [113]	Neonatal mouse, BPD from 75% O₂	AICAR (AMPK direct activator)	AMPK-ULK1–autophagy axis; autophagy-NLRP3 inflammasome link; alveolar macrophage M1/M2 polarization	↑ alveolarization (↑ RAC, ↓ MLI) and ↑ ACTA2⁺ septal-tip myofibroblasts (secondary septation); ↑ CD31 (vascular density); ↓ neutrophil and macrophage accumulation; ↑ M2 polarization in BAL. Anti-inflammatory effect is autophagy-dependent
Sudhadevi et al., 2024 [114]	Neonatal mouse 95% hyperoxia → adult follow-up	Intranasal Fingolimod (S1PR1 modulator) vs intraperitoneal fingolimod	SPHK1/S1P/S1PR1; Lysyl oxidase (LOX)/collagen cross-linking	Improved alveolarization (↓ MLI), ↓ BALF protein/total cells/neutrophils, fingolimod decreased ↓ S1PR1 and ↓ LOX in both airway and alveolar regions → ↓ collagen cross-linking; no lymphopenia with intranasal route. Adults (PN56): persistent ↓ MLI, ↓ peribronchial collagen and ↓ smooth muscle hypertrophy (myosin) ↓ airway hyperreactivity to methacholine. Notably, the ↑ S1PR1 and ↑ LOX persisted into adulthood of hyperoxia untreated mice, selectively in airways (not in alveoli), implicating airway-specific pathology in BPD sequelae. This persistent effect was reduced by the use of fingolimod
Zhong et al., 2022 [115]	Neonatal mouse, hyperoxia + lung fibroblasts	LOX inhibition (BAPN)	Lysyl oxidase (LOX)–TGF-β–Smad2/3; ECM remodeling; extracellular aldehydes	LOX inhibition →↓ aldehydes, improved ECM organization and alveolar development
Yang et al., 2025(b) [116]	Neonatal rat, hyperoxia	Prostaglandin E1	ER stress (GRP78, CHOP); apoptosis (caspase-3, Bcl-2/Bax)	↓ IL-1β/IL-6/TNF-α; ↓ caspase-3/Bax-mediated apoptosis; ↓ pulmonary edema; improved alveolar histology
Zhang et al., 2023 [117]	Neonatal rat, > 90% hyperoxia	Glutamine	ER stress (IRE1α/JNK; GRP78, CHOP); apoptosis	↓ inflammation, oxidative stress and apoptosis; ↑ RAC, ↓ MLI, RAC improved lung function (inspiratory capacity, compliance, airway resistance, tissue elasticity)
Liang et al., 2021 [118]	Neonatal rat, 90% O₂	Adenoassociated virus encoding SEMA3A	ERK/JNK; NF-κB; apoptosis and inflammation	Endogenous SEMA3A (mRNA and protein) significantly reduced in BPD model lung; AAV-SEMA3A restored body weight, lung weight, and lung-to-body weight ratio. Improved alveolar architecture: ↑ RAC, ↓ MLI, reduced inflammatory infiltrate. ↓ TUNEL⁺ apoptotic cells; ↑ Bcl-2, ↓ Bax, ↓ cleaved Caspase-3. ↓ lung IL-1β, MCP-1, TNF-α; ↓ p-NF-κB; ↓ p-ERK1/2 and ↓ p-JNK (with unchanged total ERK/JNK)
Lee et al., 2023 [119]	Neonatal rat, hyperoxia	Recombinant Hsp70/heat stimulation at birth	Hsp70-TLR4-NF-κB axis (Anti-apoptotic; anti-inflammatory)	↑ survival; ↓ early alveolar apoptosis and macrophage infiltration; ↓ histological injury
Xie et al., 2025 [120]	Neonatal mouse, hyperoxia	Sodium propionate	IL-17 pathway; epithelial apoptosis (Bax, Bcl, caspase-3)	↑ alveolarization (RAC); ↓ septal thickening; ↓ IL-17/IL-6/TNF-α; ↓ AECII apoptosis
Kryeziu et al., 2023 [121]	Neonatal rat, > 95% O₂	Quercetin	NO/cGMP signaling; TNF-α/IL-1β	↓ airway smooth muscle hyperreactivity; restored tracheal relaxation; ↓ TNF-α/IL-1β; no structural outcomes
Sopi et al., 2026 [122]	Neonatal rat, > 95% O₂	Rho-kinase inhibitors (Y-27632, fasudil)	Rho/Rho-kinase; NO–cGMP signaling (no direct measure eNOS, cGMP)	Restored tracheal smooth muscle relaxation
Chen et al., 2023 [123]	Neonatal mouse, hyperoxia	Intranasal Lactobacillus johnsonii	Gut–lung microbiota axis; angiogenesis; cytokines (IL-1β, IL-6, TNF-α on lung tissue); ZO-1/occludin (intestinal tight junctions)	↑ body weight; ↑ alveolarization (↓ MLI); ↑ pulmonary angiogenesis (↑ VEGF, ↑ vWF); ↓ pulmonary IL-1β and IL-6; ↑ intestinal ZO-1 and occludin; normalized gut microbiota (↓ Staphylococcus and Enterobacter, ↑ Lactobacillus)
Zhang et al., 2024(b) [124]	Neonatal mouse, hyperoxia + MLE 12 alveolar epithelial cells, hyperoxia at	Lactobacillus reuteri, 3-IAld, Recombinant IL-22; Anti-IL-22 neutralizing antibody	IL-22/STAT3	↑ IL-22 → STAT3 phosphorylation (in MLE-12 in vitro); ↑ alveolarization (↓ MLI, ↑ RAC); ↑ body weight; ↓ pulmonary IL-1β/IL-6/TNF-α; ↑ alveolar epithelial markers SPC and AQP5; ↑ pulmonary vascular markers FLK-1/VEGFR2 and VEGF. Mechanistic causality: (1) 3-IAld alone phenocopies L. reuteri benefit; (2) recombinant IL-22 alone is sufficient to protect; (3) anti-IL-22 abolishes L. reuteri rescue, confirming IL-22 as necessary mediator; (4) IL-22 induces STAT3 phosphorylation in MLE-12, blocked by anti-IL-22
Guillier et al., 2021 [125]	Rat, antenatal low protein diet	Nebulized curcumin	PPARγ–FABP4 axis; downregulation of profibrotic pathways: TGFβ, epithelial-mesenchymal transition, Wnt/β-catenin, Notch, Sonic Hedgehog; Anti-inflammatory; oxidative stress; NF-κB	↓ neonatal lung injury following antenatal insult; ↓ inflammation and structural damage ↓ LPD-induced abnormal alveolarization ↓ FABP4 mRNA at P11/P21 and ↓ FABP4⁺/CD206⁺ alveolar macrophage density at P21; ↑ pulmonary vessel; transcriptomic reversal of profibrotic pathways activated; well tolerated, no mortality or detectable adverse event

Search strategy and abbreviations are reported in Supplemental material

Several of these strategies converge with interventions already discussed in this review. Cytokine blockade with anti–IL-1β (anakinra) and anti-TNF-α (adalimumab), tested preclinically in chorioamnionitis-driven injury, supports the broader rationale for targeted immunomodulation. MSC and MSC-EV therapies are evolving from raw cell products toward engineered paracrine platforms, for instance MSCs overexpressing antioxidant or neurotrophic factors. The nutrient and microbiota axes are likewise represented at the bench: L-citrulline, a nitric oxide precursor, preserves alveolar and vascular growth and attenuates pulmonary hypertension in rodent hyperoxia models [28], and early-phase studies in preterm infants have characterized its pharmacokinetics and tolerability, though a dosing regimen has not been established [78, 79]. Among microbiota-targeted approaches, probiotic trials have mostly targeted other complications of prematurity, with no effect on BPD in meta-analysis [22]. A single trial (ChiCTR2400093781) with BPD as a primary endpoint awaits confirmation in independent populations [21].

A notable gap is the still-limited translational use of patient-derived platforms: nearly all signals still come from neonatal rodent hyperoxia. Hyperoxia-only models capture neither the interacting mechanisms outlined above, nor the genetic susceptibility that modulates human BPD/PLD. Patient-derived airway organoids generated from bronchoalveolar lavage of preterm infants with grade 3 BPD have very recently been reported [80], recapitulating epithelial heterogeneity, hyperoxia-induced injury responses, and dexamethasone-driven anti-inflammatory remodeling, with sex-specific differences validated against human BPD lung specimens. In addition, induced pluripotent stem cell-derived lung organoids may serve as viable substitutes when access to primary fetal tissue is limited, and represent a valuable approach to reproduce key stages of lung development. Such platforms may offer a scalable human-relevant alternative to animal models, and could potentially enable patient-stratified preclinical screening, bridging a long-standing translational gap between rodent models and the developing preterm lung [81].

Finally, gene therapy work in animal models currently seems most useful as a way to identify functional targets for downstream pharmacologic modulation, rather than as a near-term clinical option. Given the multifactorial, multi-compartment biology of PLD, lasting benefit will more plausibly emerge from combined approaches across vascular, immune and matrix programs than from correction of any single gene or pathway.

## Discussion

The development of novel therapies for BPD and PLD is shaped by significant regulatory, ethical, and methodological challenges inherent to neonatal research [126]. Neonates and infants are classified as a vulnerable population and require stringent safeguards to ensure ethical conduct, including enhanced oversight, risk minimization, and rigorous justification of potential benefits [127]. These requirements, while essential, contribute to increased complexity, cost, and duration of clinical development programs. In addition, market-related factors play a non-negligible role, as the relatively small target population limits the expected return on investment, potentially reducing industry-driven innovation in this field.

Historically, most therapeutic strategies have followed an “adult-first” development paradigm, with subsequent extrapolation to pediatric populations [126]. This approach often leads to substantial delays in the availability of new treatments for neonates, particularly in conditions such as BPD where pathophysiology is unique and not fully recapitulated in adult disease. There is growing recognition of the need to promote “pediatric-first” or “neonate-first” study designs, supported by tailored regulatory pathways that encourage early inclusion of these populations in drug development [126, 128]. Equally important is a reframing of the perceived commercial scope of these therapies. PLD should not be regarded as a condition confined to the extremely preterm infant or to early childhood: it represents a continuum spanning the full gradient of prematurity and continues to shape respiratory trajectories throughout adolescence, adulthood, and older age, with documented links to accelerated lung-function decline and COPD in later life [13, 129]. Effective interventions could therefore yield benefits well beyond the immediate target population, with implications for adult respiratory health at a societal scale—a perspective that may shift the cost-opportunity calculus for industry and support a more sustained commitment to drug development in this field [130]. Realizing this potential, however, requires a dynamic regulatory landscape, evolving to value these longer-term benefits rather than BPD alone. In addition, despite progressive efforts by regulatory agencies worldwide to mandate and incentivize pediatric drug development, harmonization across regulatory systems remains incomplete, and stronger global coordination will be essential to accelerate the safe and timely translation of innovative therapies to neonatal populations. Compounding these challenges, a definitional conundrum still affects BPD-centered outcomes. The operational nature of BPD and the coexistence of its definitions mean that both incidence and treatment-effect estimates shift depending on the definition applied in each trial [6, 7, 10]. This does not make BPD dispensable. It remains the outcome against which neonatal pulmonary trials have long been measured, and thus the endpoint that allows the therapies reviewed here to be compared with earlier ones. Disaggregated into its components of respiratory support, oxygen requirement, and pulmonary vascular disease, and combined with extended follow-up, it retains validity. Within the framework of PLD, however, therapeutic strategies can no longer be evaluated solely for their short-term efficacy, such as reductions in BPD incidence at 36 weeks PMA, but increasingly for their potential to promote physiological lung development and ultimately to modify long-term outcomes. Read as the most severe manifestation of the PLD continuum, BPD is best complemented rather than replaced by endpoints such as respiratory support at discharge, home oxygen use, pulmonary hypertension, post-discharge respiratory health care utilization, and later lung-function measures. With the exception of the 5-year follow-up of one trial, none of the therapies reviewed here has yet reported such outcomes, and the respiratory follow-up currently prespecified is not designed to capture PLD (Table 2). Preventing or attenuating BPD is expected to shift the whole spectrum of PLD, but whether that shift persists beyond infancy is something current endpoints cannot demonstrate. Later-phase and future trials should therefore consider prespecifying respiratory assessment that extends sufficiently to establish an effect on PLD, and not on BPD alone.

**Table 2:** Status and respiratory follow-up of clinical trials discussed in this review

Agent	Trial and trial phase	Population	Primary endpoint	Status	Prespecified respiratory follow-up
OHB-607 (rhIGF-1/rhIGFBP-3)	NCT03253263, Phase 2b	295, 23⁺⁰–27⁺⁶ weeks	Severe BPD or death at 36 weeks PMA	Enrolment complete	Chronic respiratory morbidity outcomes at 24 months corrected age
UC-blood derived MSCs	NCT01828957, Phase 2	66, 23–28 weeks	BPD severity	Completed	See next line
UC-blood derived MSCs	NCT01897987, follow-up of NCT01828957	59, 23–28 weeks	Composite morbidity of respiratory symptom-associated emergency department visits, hospital readmissions and oxygen use	Completed	Five-year follow-up
EXOB-001 (UC-MSC-derived EVs)	NCT06279741, Phase 1/2	36 (phase 1) and 203 (phase 2), 23⁺⁰–27⁺⁶ weeks	Safety; prevention of severe BPD	Recruiting	Lung ultrasound score and respiratory morbidity to 2 years corrected age
Zelpultide alfa (rhSP-D)	NCT04662151, Phase 1b	37, 23⁺⁰–28⁺⁶	Safety and tolerability	Completed	No prespecified long-term respiratory endpoint
Zelpultide alfa (rhSP-D)	NCT06897839, Phase 2b/3	366, 22⁺⁰–27⁺⁶	Incidence of grade 2 or grade 3 BPD or death	Recruiting	Chronic respiratory morbidity through 12 months corrected age
Anakinra	NCT05280340, Phase 1/2a	24 infants, 24⁺⁰–27⁺⁶ weeks	Safety, feasibility, pharmacokinetics	Enrollment complete, in follow-up	No prespecified long-term respiratory endpoint

## Conclusions

PLD sits at the intersection of developmental biology, lung injury and lifelong respiratory trajectory, and its therapeutic landscape is rapidly expanding. The most clinically credible interventions discussed in this review include growth-factor strategies, mesenchymal stromal cell and extracellular vesicle therapies, and cytokine blockade. Altogether, these efforts point toward a future in which PLD is managed not by targeted single-axis agents but through combinatorial, mechanism-informed approaches, tailored to the individual patient and to the specific phase of disease. Realizing this promise will require a broader recognition of PLD as a life-course condition with public health relevance well beyond the neonatal intensive care unit.

## Supplementary information

Below is the link to the electronic supplementary material.

ESM 1 (DOCX 23.4 KB)

## Data Availability

No datasets were generated or analysed during the current study.

## Abbreviations

AECII: Alveolar epithelial type II cells
BPD: Bronchopulmonary dysplasia
CASC2: Cancer susceptibility candidate 2
cGMP: Cyclic guanosine monophosphate
COPD: Chronic obstructive pulmonary disease
ELGAN: Extremely low gestational age neonate
EV: Extracellular vesicle
FUT9: Fucosyltransferase 9
GSDMD: Gasdermin D
HIF-1α: Hypoxia-inducible factor 1α
HO-1: Heme oxygenase 1
IGF-1: Insulin-like growth factor 1
IGFBP-3: Insulin-like growth factor-binding protein 3
IL-1β/-22: Interleukin-1β/-22
IRE1α: Inositol-requiring enzyme 1α
lncRNA: Long non-coding RNA
LNP: Lipid nanoparticle
LOX: Lysyl oxidase
miR: MicroRNA
MISEV: Minimal Information for Studies of Extracellular Vesicles
MMP: Matrix metalloproteinase
MSC: Mesenchymal stromal cell
NADPH: Nicotinamide adenine dinucleotide phosphate
NF-κB: Nuclear factor κB
NLRP3: NLR family pyrin domain–containing 3
NO: Nitric oxide
Nrf2: Nuclear factor erythroid 2–related factor 2
PERK: Protein kinase R–like endoplasmic reticulum kinase
PGE1: Prostaglandin E1
PLD: Prematurity-associated lung disease
PMA: Postmenstrual age
rhFUT9: Recombinant human FUT9
rhIGF-1: Recombinant human insulin-like growth factor 1
rhSP-D: Recombinant human surfactant protein D
SP-D: Surfactant protein D
sPLA2: Secretory phospholipase A2
STAT3: Signal transducer and activator of transcription 3
TGF-β: Transforming growth factor β
TLR4: Toll-like receptor 4
TNF-α: Tumor necrosis factor α
UC: Umbilical cord
VEGF: Vascular endothelial growth factor

## References

[1] World Health Organization, UNFPA, UNICEF, PMNCH (2023) Born too soon: decade of action on preterm birth. World Health Organization, Geneva

[2] Kotecha SJ, Edwards MO, Watkins WJ et al (2013) Effect of preterm birth on later FEV1: a systematic review and meta-analysis. Thorax 68:760–766. 10.1136/thoraxjnl-2012-203079 23604458

[3] Gibbons JTD, Course CW, Evans EE et al (2023) Increasing airway obstruction through life following bronchopulmonary dysplasia: a meta-analysis. ERJ Open Res 9:00046-2023. 10.1183/23120541.00046-2023 37342090 PMC10277871

[4] Bui DS, Perret JL, Walters EH et al (2022) Association between very to moderate preterm births, lung function deficits, and COPD at age 53 years: analysis of a prospective cohort study. Lancet Respir Med 10:478–484. 10.1016/S2213-2600(21)00508-7 35189074

[5] Simpson SJ, Du Berry C, Evans DJ et al (2024) Unravelling the respiratory health path across the lifespan for survivors of preterm birth. Lancet Respir Med 12:167–180. 10.1016/S2213-2600(23)00272-2 37972623

[6] Jensen EA, Dysart K, Gantz MG et al (2019) The diagnosis of bronchopulmonary dysplasia in very preterm infants. An evidence-based approach. Am J Respir Crit Care Med 200:751–759. 10.1164/rccm.201812-2348OC 30995069 PMC6775872

[7] Jobe AH, Bancalari E (2021) An all-inclusive perspective on bronchopulmonary dysplasia. J Pediatr 234:257–259. 10.1016/j.jpeds.2021.03.063 33811871

[8] Northway WHJ, Rosan RC, Porter DY (1967) Pulmonary disease following respirator therapy of hyaline-membrane disease. Bronchopulmonary dysplasia. N Engl J Med 276:357–368. 10.1056/NEJM196702162760701 5334613

[9] Baraldi E, Filippone M (2007) Chronic lung disease after premature birth. N Engl J Med 357:1946–1955. 10.1056/NEJMra067279 17989387

[10] Thébaud B, Goss KN, Laughon M et al (2019) Bronchopulmonary dysplasia Nat Rev Dis Primers 5:78. 10.1038/s41572-019-0127-7 31727986 PMC6986462

[11] Du Berry C, Nesci C, Cheong JLY et al (2022) Long-term expiratory airflow of infants born moderate-late preterm: a systematic review and meta-analysis. eClinicalMedicine 52:101597. 10.1016/j.eclinm.2022.101597 35923430 PMC9340512

[12] Zanetto L, Bonadies L, Pertoldi C et al (2025) Targeting treatable traits across the lifespan in preterm-born individuals with chronic lung disease of prematurity. Am J Respir Crit Care Med 211:1996–1998. 10.1164/rccm.202505-1157VP 40929522 PMC12619005

[13] Course CW, Bush A, Kotecha S (2026) Looking beyond bronchopulmonary dysplasia: prematurity-associated lung disease and its phenotypes. Lancet Respir Med 14:60–71. 10.1016/S2213-2600(25)00372-8 41319663

[14] Bonadies L, Zanetto L, Ferraro VA et al (2026) Bronchopulmonary dysplasia and extremely preterm birth: time for a broader perspective on long-term outcomes. Eur Respir Rev 35:250304. 10.1183/16000617.0304-2025 41951244 PMC13058737

[15] Jenkinson A, Dassios T (2026) Follow-up of neonatal chronic respiratory disease: an update based on the current evidence. Eur J Pediatr 185:61. 10.1007/s00431-025-06713-5 41501568 PMC12779740

[16] Manley BJ, Kamlin COF, Donath SM et al (2024) Intratracheal budesonide mixed with surfactant for extremely preterm infants: the PLUSS randomized clinical trial. JAMA 332:1889. 10.1001/jama.2024.17380 39527075 PMC11555571

[17] Allen MC, Donohue PK, Dusman AE (1993) The limit of viability – neonatal outcome of infants born at 22 to 25 weeks’ gestation. N Engl J Med 329:1597–1601. 10.1056/NEJM199311253292201 8179651

[18] Kneyber MCJ, Zhang H, Slutsky AS (2014) Ventilator-induced lung injury. Similarity and differences between children and adults. Am J Respir Crit Care Med 190:258–265. 10.1164/rccm.201401-0168CP 25003705 PMC4896812

[19] Alvira CM, Morty RE (2017) Can we understand the pathobiology of bronchopulmonary dysplasia? J Pediatr 190:27–37. 10.1016/j.jpeds.2017.08.041 29144252 PMC5726414

[20] Bonadies L, Zaramella P, Porzionato A et al (2020) Present and future of bronchopulmonary dysplasia. JCM 9:1539. 10.3390/jcm9051539 32443685 PMC7290764

[21] Wang D, Wang S, Wang H, Zhang X (2026) A randomized controlled trial of probiotic intervention for reducing the risk of bronchopulmonary dysplasia in preterm infants. Arch Med Res 57:103408. 10.1016/j.arcmed.2026.103408 41895129

[22] Villamor-Martínez E, Pierro M, Cavallaro G et al (2017) Probiotic supplementation in preterm infants does not affect the risk of bronchopulmonary dysplasia: a meta-analysis of randomized controlled trials. Nutrients 9:1197. 10.3390/nu9111197 29088103 PMC5707669

[23] Qu Y, Guo S, Liu Y et al (2021) Association between probiotics and bronchopulmonary dysplasia in preterm infants. Sci Rep 11:17060. 10.1038/s41598-021-96489-z 34426616 PMC8382697

[24] Zhu Y, Ding R-D (2025) Role of the gut‑lung axis in bronchopulmonary dysplasia: physiological basis, pathogenesis and immunological modulation (review). Mol Med Rep 32:1–12. 10.3892/mmr.2025.13678 PMC12447068 40937590

[25] Keever KR, Yakubenko VP, Hoover DB (2023) Neuroimmune nexus in the pathophysiology and therapy of inflammatory disorders: role of α7 nicotinic acetylcholine receptors. Pharmacol Res 191:106758. 10.1016/j.phrs.2023.106758 37028776 PMC13134767

[26] Bonadies L, Calgaro S, Stocchero M et al (2026) Early prediction of bronchopulmonary dysplasia by urinary metabolomics: a case–control study. Thorax 81:276–281. 10.1136/thorax-2025-223090 40903289 PMC13018839

[27] Strauch L, Lang P, Cheema Z et al (2026) Urine metabolomics to predict bronchopulmonary dysplasia: a single-center prospective study. Pediatr Pulmonol 61:e71644. 10.1002/ppul.71644 42125926 PMC13383976

[28] Vadivel A, Aschner JL, Rey-Parra GJ et al (2010) L-citrulline attenuates arrested alveolar growth and pulmonary hypertension in oxygen-induced lung injury in newborn rats. Pediatr Res 68:519–525. 10.1203/PDR.0b013e3181f90278 20805789 PMC3132222

[29] Kramer BW, Abman S, Daly M et al (2023) Insulin-like growth factor-1 replacement therapy after extremely premature birth: an opportunity to optimize lifelong lung health by preserving the natural sequence of lung development. Paediatr Respir Rev 48:24–29. 10.1016/j.prrv.2023.05.001 37268507

[30] Baraldi E, De Luca D, Bonadies L et al (2026) Randomised phase 2b trial of rhIGF-1/rhIGFBP-3 (OHB-607) for bronchopulmonary dysplasia prevention in preterm neonates: study protocol. BMJ Paediatr Open 10:e004196. 10.1136/bmjpo-2025-004196 41786363 PMC12970049

[31] Zhang S, Luan X, Li H, Jin Z (2022) Insulin-like growth factor-1: a potential target for bronchopulmonary dysplasia treatment (Review). Exp Ther Med 23:191. 10.3892/etm.2022.11114 35126694 PMC8794548

[32] Plosa EJ, Benjamin JT (2020) rhIGF-1 therapy: a silver bullet for bronchopulmonary dysplasia prevention? Am J Respir Crit Care Med 201:1032–1033. 10.1164/rccm.202002-0287ED 32109132 PMC7193858

[33] Ramos-Navarro C, Gregorio-Hernández R, Pérez-Pérez A et al (2025) Impact of placental pathology on the risk of bronchopulmonary dysplasia in preterm infants: the role of gestational age and sex. Eur J Pediatr 184:211. 10.1007/s00431-025-06016-9 40009187 PMC12476302

[34] Lowe J, Gillespie D, Aboklaish A et al (2024) Azithromycin therapy for prevention of chronic lung disease of prematurity (AZTEC): a multicentre, double-blind, randomised, placebo-controlled trial. Lancet Respir Med 12:608–618. 10.1016/S2213-2600(24)00079-1 38679042

[35] Albertine KH, Rebentisch A, Dawson E et al (2026) Recombinant human insulin-like growth factor 1 complex preserves lung development in mechanically ventilated preterm lambs. Am J Respir Crit Care Med 212:778–791. 10.1093/ajrccm/aamaf105 41738171 PMC13051692

[36] Ley D, Hallberg B, Hansen-Pupp I et al (2019) rhIGF-1/rhIGFBP-3 in preterm infants: a phase 2 randomized controlled trial. J Pediatr 206:56-65.e8. 10.1016/j.jpeds.2018.10.033 30471715 PMC6389415

[37] Augustine S, Avey MT, Harrison B et al (2017) Mesenchymal stromal cell therapy in bronchopulmonary dysplasia: systematic review and meta-analysis of preclinical studies. Stem Cells Transl Med 6:2079–2093. 10.1002/sctm.17-0126 29045045 PMC5702524

[38] Pittenger MF, Discher DE, Péault BM et al (2019) Mesenchymal stem cell perspective: cell biology to clinical progress. npj Regen Med 4:22. 10.1038/s41536-019-0083-6 31815001 PMC6889290

[39] Deguise M, Thébaud B (2025) The role of mesenchymal stromal cells in the treatment of bronchopulmonary dysplasia: a multi-prong approach for a heterogeneous disease. Compr Physiol 15:e70038. 10.1002/cph4.70038 40853849 PMC12377523

[40] Ahn SY, Chang YS, Sung DK et al (2013) Mesenchymal stem cells prevent hydrocephalus after severe intraventricular hemorrhage. Stroke 44:497–504. 10.1161/STROKEAHA.112.679092 23287782

[41] Mintoft A, Vallatos A, Robertson NJ (2024) Mesenchymal stromal cell therapy for hypoxic ischemic encephalopathy: future directions for combination therapy with hypothermia and/or melatonin. Semin Perinatol 48:151929. 10.1016/j.semperi.2024.151929 38902120

[42] Maltais-Bilodeau C, Henckel E, Deguise M-O et al (2025) Cell-based therapies in preclinical models of necrotizing enterocolitis: a systematic review and meta-analysis. Stem Cells Transl Med 14:szae102. 10.1093/stcltm/szae102 40036304 PMC11878585

[43] Zhu Y, Xu L, Collins JJP et al (2017) Human umbilical cord mesenchymal stromal cells improve survival and bacterial clearance in neonatal sepsis in rats. Stem Cells Dev 26:1054–1064. 10.1089/scd.2016.0329 28401804

[44] Cyr-Depauw C, Cook DP, Mižik I et al (2024) Single-cell RNA sequencing reveals repair features of human umbilical cord mesenchymal stromal cells. Am J Respir Crit Care Med 210:814–827. 10.1164/rccm.202310-1975OC 38564376

[45] Cyr-Depauw C, Mižik I, Cook DP et al (2025) Single-cell RNA sequencing to guide autologous preterm cord mesenchymal stromal cell therapy. Am J Respir Crit Care Med 211:391–406. 10.1164/rccm.202403-0569OC 39586004

[46] Chang YS, Ahn SY, Yoo HS et al (2014) Mesenchymal stem cells for bronchopulmonary dysplasia: phase 1 dose-escalation clinical trial. J Pediatr 164:966-972.e6. 10.1016/j.jpeds.2013.12.011 24508444

[47] Ahn SY, Chang YS, Kim JH et al (2017) Two-year follow-up outcomes of premature infants enrolled in the phase I trial of mesenchymal stem cells transplantation for bronchopulmonary dysplasia. J Pediatr 185:49-54.e2. 10.1016/j.jpeds.2017.02.061 28341525

[48] Pierro M, Thébaud B, Soll R (2017) Mesenchymal stem cells for the prevention and treatment of bronchopulmonary dysplasia in preterm infants. Cochrane Database of Systematic Reviews 2017:. 10.1002/14651858.CD011932.pub2 PMC6485972 29125893

[49] Ahn SY, Chang YS, Lee MH et al (2023) Five-year follow-up of phase II trial of stromal cells for bronchopulmonary dysplasia. Thorax 78:1105–1110 37604693 10.1136/thorax-2022-219622

[50] Dominici M, Le Blanc K, Mueller I et al (2006) Minimal criteria for defining multipotent mesenchymal stromal cells. The International Society for Cellular Therapy position statement. Cytotherapy 8:315–317. 10.1080/14653240600855905 16923606

[51] Welsh JA, Goberdhan DCI, O’Driscoll L et al (2024) Minimal information for studies of extracellular vesicles (MISEV2023): from basic to advanced approaches. J of Extracellular Vesicle 13:e12404. 10.1002/jev2.12404 PMC10850029 38326288

[52] Saneh H, Wanczyk H, Walker J, Finck C (2025) Stem cell-derived extracellular vesicles: a potential intervention for bronchopulmonary dysplasia. Pediatr Res 97:497–509. 10.1038/s41390-024-03471-2 39251881 PMC12014501

[53] Lesage F, Thébaud B (2022) Mesenchymal stromal cell-derived extracellular vesicles for neonatal lung disease: tiny particles, major promise, rigorous requirements for clinical translation. Cells 11:1176. 10.3390/cells11071176 35406742 PMC8997376

[54] Ransom MA, Blatt AM, Pua HH, Sucre JMS (2024) The emerging role of extracellular vesicles in bronchopulmonary dysplasia. Am J Physiol Lung Cell Mol Physiol 326:L517–L523. 10.1152/ajplung.00244.2023 38469633 PMC11380955

[55] Willis GR, Fernandez-Gonzalez A, Anastas J et al (2018) Mesenchymal stromal cell exosomes ameliorate experimental bronchopulmonary dysplasia and restore lung function through macrophage immunomodulation. Am J Respir Crit Care Med 197:104–116. 10.1164/rccm.201705-0925OC 28853608 PMC5765387

[56] Willis GR, Fernandez‐Gonzalez A, Reis M et al (2020) Mesenchymal stromal cell‐derived small extracellular vesicles restore lung architecture and improve exercise capacity in a model of neonatal hyperoxia‐induced lung injury. J of Extracellular Vesicle 9:1790874. 10.1080/20013078.2020.1790874 PMC7480622 32939235

[57] Porzionato A, Zaramella P, Dedja A et al (2019) Intratracheal administration of clinical-grade mesenchymal stem cell-derived extracellular vesicles reduces lung injury in a rat model of bronchopulmonary dysplasia. Am J Physiol Lung Cell Mol Physiol 316:L6–L19. 10.1152/ajplung.00109.2018 30284924

[58] Porzionato A, Zaramella P, Dedja A et al (2021) Intratracheal administration of mesenchymal stem cell-derived extracellular vesicles reduces lung injuries in a chronic rat model of bronchopulmonary dysplasia. Am J Physiol Lung Cell Mol Physiol 320:L688–L704. 10.1152/ajplung.00148.202 33502939

[59] Bisaccia P, Magarotto F, D’Agostino S et al (2024) Extracellular vesicles from mesenchymal umbilical cord cells exert protection against oxidative stress and fibrosis in a rat model of bronchopulmonary dysplasia. Stem Cells Transl Med 13:43–59. 10.1093/stcltm/szad070 37963808 PMC10785219

[60] Abele AN, Taglauer ES, Almeda M et al (2022) Antenatal mesenchymal stromal cell extracellular vesicle treatment preserves lung development in a model of bronchopulmonary dysplasia due to chorioamnionitis. Am J Physiol Lung Cell Mol Physiol 322:L179–L190. 10.1152/ajplung.00329.2021 34878940 PMC8782653

[61] Lithopoulos MA, Strueby L, O’Reilly M et al (2022) Pulmonary and neurologic effects of mesenchymal stromal cell extracellular vesicles in a multifactorial lung injury model. Am J Respir Crit Care Med 205:1186–1201. 10.1164/rccm.202012-4520OC 35286238

[62] Albertine KH, Rebentisch A, Dawson E et al (2024) Mesenchymal stromal cell extracellular vesicles improve lung development in mechanically ventilated preterm lambs. Am J Physiol Lung Cell Mol Physiol 326:L770–L785. 10.1152/ajplung.00349.2023 38563994 PMC11380989

[63] Arroyo R, Kingma PS (2021) Surfactant protein D and bronchopulmonary dysplasia: a new way to approach an old problem. Respir Res 22:141. 10.1186/s12931-021-01738-4 33964929 PMC8105703

[64] De Luca D, Arroyo R, Foligno S et al (2023) Early life surfactant protein-D levels in bronchoalveolar lavage fluids of extremely preterm neonates. Am J Physiol Lung Cell Mol Physiol 325:L411–L418. 10.1152/ajplung.00079.2023 37489844

[65] Kotecha S, Davies PL, Clark HW, McGreal EP (2013) Increased prevalence of low oligomeric state surfactant protein D with restricted lectin activity in bronchoalveolar lavage fluid from preterm infants. Thorax 68:460–467. 10.1136/thoraxjnl-2012-202729 23390139

[66] Jain VG, Parikh NA, Rysavy MA et al (2024) Histological chorioamnionitis increases the risk of bronchopulmonary dysplasia. Am J Respir Crit Care Med 209:1272–1275. 10.1164/rccm.202311-2084LE 38416515 PMC11146533

[67] De Luca D, Foligno S, Autilio C et al (2022) Secretory phospholipase A2 expression and activity in preterm clinical chorioamnionitis with fetal involvement. Am J Physiol Lung Cell Mol Physiol 323:L121–L128. 10.1152/ajplung.00516.2021 35762614

[68] Beresford MW, Shaw NJ (2003) Bronchoalveolar lavage surfactant protein A, B, and D concentrations in preterm infants ventilated for respiratory distress syndrome receiving natural and synthetic surfactants. Pediatr Res 53:663–670. 10.1203/01.PDR.0000054653.89527.F8 12612206

[69] De Luca D, Shankar-Aguilera S, Autilio C et al (2020) Surfactant-secreted phospholipase A_2_ interplay and respiratory outcome in preterm neonates. Am J Physiol Lung Cell Mol Physiol 319:L95–L104. 10.1152/ajplung.00462.2019 32401671

[70] Sorensen GL, Dahl M, Tan Q et al (2014) Surfactant protein-D–encoding gene variant polymorphisms are linked to respiratory outcome in premature infants. J Pediatr 165:683–689. 10.1016/j.jpeds.2014.05.042 25015576

[71] Vinod S, Gow A, Weinberger B et al (2019) Serum surfactant protein D as a marker for bronchopulmonary dysplasia. J Matern Fetal Neonatal Med 32:815–819. 10.1080/14767058.2017.1392506 29025303

[72] Green EA, Metz D, Galinsky R et al (2022) Anakinra pilot – a clinical trial to demonstrate safety, feasibility and pharmacokinetics of interleukin 1 receptor antagonist in preterm infants. Front Immunol 13:1022104. 10.3389/fimmu.2022.1022104 36389766 PMC9647081

[73] Eldredge LC, Creasy RS, Presnell S et al (2019) Infants with evolving bronchopulmonary dysplasia demonstrate monocyte-specific expression of IL-1 in tracheal aspirates. Am J Physiol Lung Cell Mol Physiol 317:L49–L56. 10.1152/ajplung.00060.2019 30969811

[74] Nold MF, Mangan NE, Rudloff I et al (2013) Interleukin-1 receptor antagonist prevents murine bronchopulmonary dysplasia induced by perinatal inflammation and hyperoxia. Proc Natl Acad Sci U S A 110:14384–14389. 10.1073/pnas.1306859110 23946428 PMC3761642

[75] Royce SG, Nold MF, Bui C et al (2016) Airway remodeling and hyperreactivity in a model of bronchopulmonary dysplasia and their modulation by IL-1 receptor antagonist. Am J Respir Cell Mol Biol 55:858–868. 10.1165/rcmb.2016-0031OC 27482635

[76] Bui CB, Kolodziej M, Lamanna E et al (2019) Interleukin-1 receptor antagonist protects newborn mice against pulmonary hypertension. Front Immunol 10:1480. 10.3389/fimmu.2019.01480 31354700 PMC6637286

[77] De Rose DU, Cesaroni GM, Campi F et al (2026) Respiratory effects of off label anakinra in critically ill neonates: a 15-patient observational cohort study. Front Pediatr 14:1806329. 10.3389/fped.2026.1806329 42220990 PMC13219249

[78] Fike CD, Avachat C, Birnbaum AK et al (2023) Pharmacokinetics of l-citrulline in neonates at risk of developing bronchopulmonary dysplasia-associated pulmonary hypertension. Pediatr Drugs 25:87–96. 10.1007/s40272-022-00542-x PMC10039462 36316628

[79] Fike CD, Summar M, Aschner JL (2014) L-citrulline provides a novel strategy for treating chronic pulmonary hypertension in newborn infants. Acta Paediatr 103:1019–1026. 10.1111/apa.12707 24862864 PMC4209175

[80] Sonti S, Cantu A, Cantu Guttierez M et al (2026) Patient-derived lung organoids from bronchoalveolar lavage capture epithelial heterogeneity and disease biology in bronchopulmonary dysplasia. Redox Biol 90:104057. 10.1016/j.redox.2026.104057 41621243 PMC12882664

[81] Zanetto L, Bonadies L, Moll-Diaz R et al (2025) Lung organoids: a new frontier in neonatology and paediatric respiratory medicine. Eur Respir Rev 34:240255. 10.1183/16000617.0255-2024 40769535 PMC12340526

[82] León Silva AH, Tian R, Ballengee S et al (2025) Caspase-1 inhibition mitigates neonatal hyperoxia-induced vascular and cardiopulmonary inflammation in neonatal rats. Clin Sci (Lond) 139:1611–1627. 10.1042/CS20242275 41231039 PMC12751044

[83] Feng Y, Chen D-Z, Cai H, Zhang Q-Y (2026) Dexmedetomidine attenuates hyperoxia-induced lung injury in BPD mice via regulation of the mitophagy–NLRP3–pyroptosis pathway. Arch Biochem Biophys 778:110748. 10.1016/j.abb.2026.110748 41581638

[84] Zhang J, Zhou L, Xu H et al (2026) Betaine improves hyperoxic lung injury through downregulating pulmonary macrophage pyroptosis in newborn mice. Pediatr Res 99:1613–1620. 10.1038/s41390-025-04364-8 40973747

[85] Yang X, Luo G, Lou F (2025) Nesfatin-1 suppresses inflammation in bronchopulmonary dysplasia by regulating HMGB-1/TLR4/p65/NLRP3 signaling pathway. Discov Med 37:933. 10.24976/Discov.Med.202537196.83 40415368

[86] Zhang Y, Ren X, Zhang L et al (2024) Hydrogen gas inhalation ameliorates LPS-induced BPD by inhibiting inflammation via regulating the TLR4–NFκB–IL6/NLRP3 signaling pathway in the placenta. Eur J Med Res 29:285. 10.1186/s40001-024-01874-9 38745325 PMC11092067

[87] Hao R, Li Y, Li J et al (2025) Nintedanib alleviates hyperoxia-induced lung injury via targeting NF-κB signalling pathway in rat model of bronchopulmonary dysplasia. Folia Histochem Cytobiol 63:79–87. 10.5603/fhc.105218 40421818

[88] Aslan M, Gokce IK, Turgut H et al (2023) Molsidomine decreases hyperoxia-induced lung injury in neonatal rats. Pediatr Res 94:1341–1348. 10.1038/s41390-023-02643-w 37179436

[89] Deng J, Wang S-H, Zheng X-M, Tang Z-M (2022) Calcitonin gene-related peptide attenuates hyperoxia-induced oxidative damage in alveolar epithelial type II cells through regulating viability and transdifferentiation. Inflammation 45:863–875. 10.1007/s10753-021-01591-z 34988756

[90] Guzmán-Navarro G, de León MB, Martín-Estal I et al (2021) Prenatal indole-3-carbinol administration activates aryl hydrocarbon receptor-responsive genes and attenuates lung injury in a bronchopulmonary dysplasia model. Exp Biol Med (Maywood) 246:695–706. 10.1177/1535370220963789 33148012 PMC7988727

[91] Montgomery HD, Zhang MA, Zimmerman E, Helms MN (2026) Flavin adenine dinucleotide increases antioxidant availability and protects neonatal C57Bl6 lungs from high oxygen induced lung injury. Am J Physiol Cell Physiol 330:C1057–C1066. 10.1152/ajpcell.00102.2026 41811728 PMC13128293

[92] Pini A, Nardini P, Zizi V et al (2026) β(3)-adrenoceptor agonism exerts lung protection in a rat model of bronchopulmonary dysplasia. Br J Pharmacol 183:2497–2517. 10.1111/bph.70333 41577340

[93] Reçica R, Kryeziu I, Thaçi Q et al (2024) Protective effects of resveratrol against airway hyperreactivity, oxidative stress, and lung inflammation in a rat pup model of bronchopulmonary dysplasia. Physiol Res 73:239–251. 10.33549/physiolres.935239 38710061 PMC11081184

[94] Zhao Z-W, Lin X-X, Guo Y-Z et al (2023) Irisin alleviates hyperoxia-induced bronchopulmonary dysplasia through activation of Nrf2/HO-1 pathway. Peptides 170:171109. 10.1016/j.peptides.2023.171109 37804931

[95] Chu X, Zhang X, Weng B et al (2023) Erythromycin attenuates hyperoxia induced lung injury by enhancing GSH expression and inhibiting expression of inflammatory cytokines. Fetal Pediatr Pathol 42:766–774. 10.1080/15513815.2023.2223722 37341579

[96] Ozdemir R, Gokce IK, Tekin S et al (2022) The protective effects of apocynin in hyperoxic lung injury in neonatal rats. Pediatr Pulmonol 57:109–121. 10.1002/ppul.25707 34581514

[97] Yang X, Jin Z, Wang X et al (2023) Nesfatin-1 alleviates hyperoxia-induced lung injury in newborn mice by inhibiting oxidative stress through regulating SIRT1/PGC-1α pathway. Cytokine 169:156239. 10.1016/j.cyto.2023.156239 37301191

[98] Chang JL, Gong J, Rizal S et al (2022) Upregulating carnitine palmitoyltransferase 1 attenuates hyperoxia-induced endothelial cell dysfunction and persistent lung injury. Respir Res 23:205. 10.1186/s12931-022-02135-1 35964084 PMC9375342

[99] Ding KL, Smith C, Seedorf G, Abman SH (2025) Nintedanib preserves lung growth and prevents pulmonary hypertension in a hyperoxia-induced lung injury model. Pediatr Res 97:1676–1683. 10.1038/s41390-024-03562-0 39394424 PMC12119323

[100] Huang L-T, Chou H-C, Chen C-M (2021) Roxadustat attenuates hyperoxia-induced lung injury by upregulating proangiogenic factors in newborn mice. Pediatr Neonatol 62:369–378. 10.1016/j.pedneo.2021.03.012 33865748

[101] Xiang X, Zhou L, Lin Z et al (2022) Metformin regulates macrophage polarization via the Shh signaling pathway to improve pulmonary vascular development in bronchopulmonary dysplasia. IUBMB Life 74:259–271. 10.1002/iub.2588 34910358

[102] Daniel KL, Gaudet C, Hamraghani A et al (2025) Sodium nitrite prevents impaired postnatal alveolar development. Am J Physiol Lung Cell Mol Physiol 328:L586–L602. 10.1152/ajplung.00324.2024 40059632

[103] Sugar SS, Heyob KM, Cheng X et al (2021) Perinatal inflammation alters histone 3 and histone 4 methylation patterns: effects of MiR-29b supplementation. Redox Biol 38:101783. 10.1016/j.redox.2020.101783 33202301 PMC7677713

[104] Ji L, Liu Z, Dong C et al (2021) LncRNA CASC2 targets CAV1 by competitively binding with microRNA-194-5p to inhibit neonatal lung injury. Exp Mol Pathol 118:104575. 10.1016/j.yexmp.2020.104575 33212124

[105] Heyob KM, Khuhro Z, Khan AQ et al (2023) Effects of DNA methylase inhibitors in a murine model of severe BPD. Respir Physiol Neurobiol 313:104060. 10.1016/j.resp.2023.104060 37031925 PMC11736813

[106] Song H, Hao Q, Li J et al (2026) Overexpression of peroxiredoxin 6 in bone marrow-derived mesenchymal stem cells amplifies their in vivo effect on bronchopulmonary dysplasia by increasing mesencephalic astrocyte-derived neurotrophic factor secretion. Toxicol Appl Pharmacol 506:117624. 10.1016/j.taap.2025.117624 41176133

[107] Chaubey S, Nader YM, Shah D et al (2021) α1,3-Fucosyltransferase-IX, an enzyme of pulmonary endogenous lung stem cell marker SSEA-1, alleviates experimental bronchopulmonary dysplasia. Pediatr Res 89:1126–1135. 10.1038/s41390-020-0891-9 32303051

[108] Toth A, Steinmeyer S, Kannan P et al (2022) Inflammatory blockade prevents injury to the developing pulmonary gas exchange surface in preterm primates. Sci Transl Med 14:eabl8574. 10.1126/scitranslmed.abl8574 35353543 PMC9082785

[109] Xu Q, Li P, Gao J, Wang W (2026) Therapeutic targeting of oxidative-inflammatory crosstalk by apelin-12 in neonatal hyperoxia-induced lung injury. Tissue Cell 99:103285. 10.1016/j.tice.2025.103285 41494360

[110] Graumuller F, Rajendran DT, Li Y et al (2026) Attenuation of experimental bronchopulmonary dysplasia by dimethyl fumarate. Pediatr Res. 10.1038/s41390-026-05039-8 42143183

[111] Tayman C, Çakır U, Akduman H et al (2021) The therapeutic effect of apocynin against hyperoxy and inflammation-induced lung injury. Int Immunopharmacol 101:108190. 10.1016/j.intimp.2021.108190 34607228

[112] Das P, Acharya S, Prahaladan VM et al (2021) Chitin-derived AVR-48 prevents experimental bronchopulmonary dysplasia (BPD) and BPD-associated pulmonary hypertension in newborn mice. Int J Mol Sci 22:8547. 10.3390/ijms22168547 34445253 PMC8395179

[113] Soni S, Jiang Y, Zhang L et al (2023) AMPK-driven macrophage responses are autophagy dependent in experimental bronchopulmonary dysplasia. Am J Respir Cell Mol Biol 68:279–287. 10.1165/rcmb.2022-0282OC 36306501 PMC9989474

[114] Sudhadevi T, Annadi A, Basa P et al (2024) Fingolimod, a sphingosine-1-phosphate receptor modulator, prevents neonatal bronchopulmonary dysplasia and subsequent airway remodeling in a murine model. J Appl Physiol (1985) 137:1231–1242. 10.1152/japplphysiol.00311.2024 39262336 PMC11563639

[115] Zhong Y, Mahoney RC, Khatun Z et al (2022) Lysyl oxidase regulation and protein aldehydes in the injured newborn lung. Am J Physiol Lung Cell Mol Physiol 322:L204–L223. 10.1152/ajplung.00158.2021 34878944 PMC8794022

[116] Yang Z, Song J, Guo J et al (2025) Effects of PGE1 on the ERS pathway in neonatal rats with hyperoxic lung injury. Pediatr Res 97:835–842. 10.1038/s41390-024-03381-3 39014239

[117] Zhang S, Li X, Yuan T et al (2023) Glutamine inhibits inflammation, oxidative stress, and apoptosis and ameliorates hyperoxic lung injury. J Physiol Biochem 79:613–623. 10.1007/s13105-023-00961-5 37145351

[118] Liang Z, Zhang X, Liu Y et al (2021) SEMA3A protects against hyperoxia-induced lung injury in a bronchopulmonary dysplasia model of newborn rat by inhibiting ERK pathway. Allergol Immunopathol (Madr) 49:8–15. 10.15586/aei.v49i6.478 34761651

[119] Lee C-H, Su T-C, Lee M-S et al (2023) Heat shock protein 70 protects the lungs from hyperoxic injury in a neonatal rat model of bronchopulmonary dysplasia. PLoS ONE 18:e0285944. 10.1371/journal.pone.0285944 37200358 PMC10194897

[120] Xie A, Qian W, Ye D et al (2025) Sodium propionate protects against bronchopulmonary dysplasia by inhibiting IL-17-mediated apoptosis of alveolar epithelial cells. Sci Rep 15:11722. 10.1038/s41598-025-94794-5 40188136 PMC11972331

[121] Kryeziu I, Reçica S, Thaçi Q et al (2023) Quercetin supplementation attenuates airway hyperreactivity and restores airway relaxation in rat pups exposed to hyperoxia. Exp Biol Med (Maywood) 248:1492–1499. 10.1177/15353702231199468 37837396 PMC10666724

[122] Sopi RB, Thaçi Q, Raffay TM, Beqiraj-Zeqiraj Q (2026) Inhibition of Rho-kinase restores impaired relaxation of airway smooth muscle in rat pups exposed to neonatal hyperoxia. Physiol Rep 14:e70728. 10.14814/phy2.70728 41546165 PMC12811411

[123] Chen C-M, Yang Y-CSH, Chou H-C, Lin S (2023) Intranasal administration of *Lactobacillus johnsonii* attenuates hyperoxia-induced lung injury by modulating gut microbiota in neonatal mice. J Biomed Sci 30:57. 10.1186/s12929-023-00958-8 37517995 PMC10388480

[124] Zhang M, Li D, Sun L et al (2024) *Lactobacillus reuteri* alleviates hyperoxia-induced BPD by activating IL-22/STAT3 signaling pathway in neonatal mice. Mediators Inflamm. 10.1155/mi/4965271 39687635 PMC11649352

[125] Guillier C, Carrière D, Pansiot J et al (2021) Nebulized curcumin protects neonatal lungs from antenatal insult in rats. Am J Physiol Lung Cell Mol Physiol 321:L545–L552. 10.1152/ajplung.00195.2021 34159801

[126] Massaro AN, Fletcher EP, Madabushi R et al (2025) A pediatric research imperative: addressing neonates in drug development through understanding neonatal clinical pharmacology. J Pediatr Pharmacol Ther 30:8–16 39935560 10.5863/1551-6776-30.1.8 PMC11809530

[127] Roth-Cline M, Nelson R (2015) Ethical considerations in conducting pediatric and neonatal research in clinical pharmacology. Curr Pharm Des 21:5619–5635 26323417 10.2174/1381612821666150901105146

[128] De Luca D, Modi N, Davis P et al (2025) The Lancet Child & adolescent health commission on the future of neonatology. Lancet Child Adolesc Health 9:578–612. 10.1016/S2352-4642(25)00106-3 40580970

[129] Global Initiative for Chronic Obstructive Lung Disease (2026) Global strategy for prevention, diagnosis and management of COPD: 2026 report. https://goldcopd.org/2026-gold-report-and-pocket-guide/. Accessed 16 August 2026

[130] Álvarez-Fuente M, Arruza L, Muro M et al (2017) The economic impact of prematurity and bronchopulmonary dysplasia. Eur J Pediatr 176:1587–1593 28889192 10.1007/s00431-017-3009-6

