# Supplementary material for: Bronchopulmonary dysplasia and prematurity-associated lung disease: emerging therapies to improve lifelong respiratory outcomes
Source: Eur J Pediatr. 2026 Sep 5;185(9):721. doi: 10.1007/s00431-026-07366-8 (PMC13546366; doi:10.1007/s00431-026-07366-8)
Supplement: Supplementary file 1 [file 431_2026_7366_MOESM1_ESM.docx]

**Supplemental material**

**European Journal of Pediatrics**

**Doi: 10.1007/s00431-026-07366-8**

**S1. Search strategy for table 1**

Full-text available studies from the following search strategy (searched on May 16th 2026): ("bronchopulmonary dysplasia"[MeSH Terms] OR bronchopulmonary dysplasia[tiab] OR BPD[tiab] OR "chronic lung disease of prematurity"[tiab] OR "prematurity-associated lung disease"[tiab] OR "prematurity associated lung disease"[tiab] OR PALD[tiab] OR "new bronchopulmonary dysplasia"[tiab] OR "neonatal lung injury"[tiab] OR "hyperoxia-induced lung injury"[tiab] OR "impaired alveolarization"[tiab] OR "alveolar simplification"[tiab]) AND ("Animals"[MeSH Terms] NOT "Humans"[MeSH Terms]) AND (animal model*[tiab] OR experimental model*[tiab] OR murine[tiab] OR mouse[tiab] OR mice[tiab] OR rat[tiab] OR rats[tiab] OR rabbit*[tiab] OR lamb*[tiab] OR sheep[tiab] OR piglet*[tiab] OR swine[tiab] OR baboon*[tiab] OR primate*[tiab] OR hyperoxia[tiab]) AND (therap*[tiab] OR treatment*[tiab] OR intervention*[tiab] OR prevent*[tiab] OR rescue[tiab] OR regenerat*[tiab] OR repar*[tiab] OR "stem cell*"[tiab] OR "mesenchymal stromal cell*"[tiab] OR MSC[tiab] OR extracellular vesicle*[tiab] OR exosome*[tiab] OR "conditioned medium"[tiab] OR angiogenic[tiab] OR anti-inflammatory[tiab] OR antioxidant*[tiab] OR growth factor*[tiab] OR cytokine*[tiab] OR gene therap*[tiab] OR miRNA[tiab] OR microRNA[tiab] OR siRNA[tiab] OR nanoparticle*[tiab] OR biologic*[tiab] OR pharmacologic*[tiab] OR drug*[tiab]) NOT (review[Publication Type] OR systematic review[Publication Type] OR meta-analysis[Publication Type]) filter last 5 years.

**S2. Abbreviations for table 1**

Abbreviations: 3-IAld, 3-indole aldehyde; AAV, adeno-associated virus; ACTA2, α-actin 2; AECI/AECII (AT1/ATII), alveolar epithelial type I/II cells; AhR, aryl hydrocarbon receptor; AICAR, 5-aminoimidazole-4-carboxamide ribonucleotide; AMPK, AMP-activated protein kinase; Ang2, angiopoietin-2; AQP5, aquaporin-5; αSMA, α-smooth muscle actin; BAL/BALF, bronchoalveolar lavage / lavage fluid; Bax, Bcl-2–associated X protein; Bcl-2, B-cell lymphoma 2; (BM-)MSC, (bone marrow–derived) mesenchymal stromal cell; BPD, bronchopulmonary dysplasia; CASC2, cancer susceptibility candidate 2 (lncRNA); CAV1, caveolin 1; CCL/CXCL, C-C / C-X-C motif chemokine ligand; CD31, cluster of differentiation 31 (PECAM-1); cGMP, cyclic guanosine monophosphate; CHOP, C/EBP homologous protein; CPT1a, carnitine palmitoyltransferase 1a; Cyp1a1, cytochrome P450 1A1; DNA, deoxyribonucleic acid; DNMT, DNA methyltransferase; ECM, extracellular matrix; EMT, epithelial–mesenchymal transition; eNOS, endothelial nitric oxide synthase; ER, endoplasmic reticulum; ERK, extracellular signal–regulated kinase; FABP4, fatty acid–binding protein 4; FAD, flavin adenine dinucleotide; FLK-1, fetal liver kinase 1 (VEGFR2); FOXO1, forkhead box O1; FUT9, fucosyltransferase 9; GFP, green fluorescent protein; Gli1, glioma-associated oncogene 1; GPx, glutathione peroxidase; GRP78, glucose-regulated protein 78; GSDMD, gasdermin D; GSH/GSSG, reduced/oxidized glutathione; Gsta1, glutathione S-transferase α-1; H₂, molecular hydrogen; H3K4me3/H3K27me3/H4K20me3, histone H3 lysine 4/27 and H4 lysine 20 trimethylation; HERP, homocysteine-induced ER protein; Hes1, hairy and enhancer of split 1; HIF-1α, hypoxia-inducible factor 1α; HMGB1, high-mobility group box 1; HO-1, heme oxygenase 1; Hsp70, heat shock protein 70; IL-1β/-6/-10/-17/-18/-22, interleukin-1β/-6/-10/-17/-18/-22; iNO, inhaled nitric oxide; iNOS, inducible nitric oxide synthase; IRE1α, inositol-requiring enzyme 1α; JNK, c-Jun N-terminal kinase; Ki67, proliferation marker Ki-67; KO, knockout; lncRNA, long non-coding RNA; LNP, lipid nanoparticle; LOX, lysyl oxidase; LPD, low-protein diet; LPS, lipopolysaccharide; MANF, mesencephalic astrocyte-derived neurotrophic factor; MCP-1, monocyte chemoattractant protein 1; MDA, malondialdehyde; miR, microRNA; MLI, mean linear intercept; MMP-9, matrix metalloproteinase 9; MPO, myeloperoxidase; mTOR, mammalian target of rapamycin; NADPH, nicotinamide adenine dinucleotide phosphate; NaNO₂, sodium nitrite; NF-κB, nuclear factor κB; NLRP1/NLRP3, NLR family pyrin domain–containing 1/3; NO, nitric oxide; NOx, nitric oxide metabolites; Nqo1, NAD(P)H quinone dehydrogenase 1; Nrf2, nuclear factor erythroid 2–related factor 2; PCNA, proliferating cell nuclear antigen; PDGF/PDGFR(α), platelet-derived growth factor / receptor (α); PGC-1α, PPARγ coactivator 1α; PH, pulmonary hypertension; PINK1, PTEN-induced kinase 1; PN, postnatal day; PPARγ, peroxisome proliferator–activated receptor γ; PRDX6, peroxiredoxin 6; PRMT1/PRMT5, protein arginine methyltransferase 1/5; RAC, radial alveolar count; RDS, respiratory distress syndrome; ROS, reactive oxygen species; RVH: right ventricular hypertrophy, S1P/S1PR1, sphingosine-1-phosphate / S1P receptor 1; SEMA3A, semaphorin 3A; sGC, soluble guanylate cyclase; sh-CAV1, short-hairpin CAV1; Shh, Sonic Hedgehog; SIRT1, sirtuin 1; Smad2/3, small mothers against decapentaplegic 2/3; SOD, superoxide dismutase; SP-B/SP-C, surfactant protein B/C; SpO₂, peripheral oxygen saturation; SPHK1, sphingosine kinase 1; Src, Src family tyrosine kinase; SSEA-1, stage-specific embryonic antigen 1; STAT3, signal transducer and activator of transcription 3; TGF-β, transforming growth factor β; TLR4, Toll-like receptor 4; TNF-α, tumor necrosis factor α; TUNEL, terminal deoxynucleotidyl transferase dUTP nick end labeling; ULK1, Unc-51–like autophagy activating kinase 1; VEGF/VEGF-D/VEGFR2, vascular endothelial growth factor / VEGF-D / VEGF receptor 2; vWF, von Willebrand factor; WNT, wingless/Int-1; ZO-1, zonula occludens-1.
